# Survivorship and food consumption of immatures and adults of *Apis mellifera* and *Scaptotrigona bipunctata* exposed to genetically modified eucalyptus pollen

**DOI:** 10.1007/s11248-023-00343-z

**Published:** 2023-04-07

**Authors:** Charles F. dos Santos, Jenifer D. Ramos, Fernanda G. de Carvalho, Andressa L. Dorneles, Thais R. D. Menezes, Ana Cristina Pinheiro, Betina Blochtein

**Affiliations:** 1 Escola de Ciências da Saúde e da Vida, Pontifícia Universidade Católica do Rio Grande do Sul, Av. Ipiranga, 6681, Porto Alegre, RS 90619-900 Brazil; 2 Suzano S.A. (FuturaGene - Biotech Division), Itapetininga, SP 18207-780 Brazil

**Keywords:** Biotechnology, Biosafety, GMO, Pollinator, Risk assessment, Social bees

## Abstract

*Eucalyptus* comprises the largest planted area of cultivated production forest in Brazil. Genetic modification (GM) of eucalyptus can provide additional characteristics for increasing productivity and protecting wood yield, as well as potentially altering fiber for a diversity of industrial uses. However, prior to releasing a new GM plant, risk assessments studies with non-target organisms must be undertaken. Bees are prominent biological models since they play an important role in varied ecosystems, including for *Eucalyptus* pollination. The main goal of this study was to evaluate whether a novel event (*Eucalyptus* 751K032), which carries the *cp4-epsps* gene that encodes the protein CP4-EPSPS and *nptII* gene that encodes the protein NPTII, might adversely affect honey bees (*Apis mellifera*) and stingless bees (*Scaptotrigona bipunctata*). The experiments were performed in southern Brazil, as follows: (i) larvae and adults were separately investigated, (ii) three or four different pollen diets were offered to bees, depending on larval or adult status, and (iii) two biological attributes, *i.e.*, survivorship of larvae and adults and food intake by adults were evaluated. The diets were prepared with pollen from GM *Eucalyptus* 751K032; pollen from conventional *Eucalyptus* clone FGN-K, multifloral pollen or pure larval food. The insecticide dimethoate was used to evaluate the sensitivity of bees to toxic substances. Datasets were analyzed with Chi-square test, survival curves and repeated measures ANOVA. Results indicated no evidence of adverse effects of Eucalyptus pollen 751K032 on either honey bees or stingless bees assessed here. Therefore, the main findings suggest that the novel event may be considered harmless to these organisms since neither survivorship nor food consumption by bees were affected by it.

## Introduction

Biotechnology in the form of genetic engineering has been widely employed into multiple commercial plantations around the world (Park et al. 2011). Such improvements have been targeted at meeting growing demands for timber, cellulose, paper, and biofuel (Harfouche et al. 2011; Meilan et al. 2012; Chang et al. 2018; Arpaia et al. 2021). One of the most important cultivated tree that has being targeted with genetic engineering is the *Eucalyptus* genus (Grattapaglia and Kirst 2008; Harfouche et al. 2011; Lucas et al. 2017; Silva et al. 2017). *Eucalyptus* covers the largest planted area of cultivated production forest in Brazil (de Oliveira et al. 2020; Assad et al. 2022). The current area of 7.5 million hectares represents approximately 75% of the entire planted forest area in the country (IBGE 2020; IBA 2021). Genetic modification of *Eucalyptus* provides an addition to conventional tree breeding approaches for significantly increasing and protecting growth, as well as potentially providing improved fiber for a diversity of industrial uses. Currently, *Eucalyptus* provides raw materials for diverse economic sectors (paper/cardboard/packaging, cellulose, furniture, civil construction, resins, and bio-products), while also generating energy for agriculture and steel production (de Oliveira et al. 2020; Assad et al. 2022).

Prior to releasing genetically modified plants in the environment, a risk assessment to identify and evaluate exposure of non-target organisms to the new GM crop along its life cycle must be conducted (Romeis et al. 2008, 2011; Hilbeck et al. 2011; Ricroch et al. 2018). In Brazil, the legislation specifying safety norms and inspection mechanisms for activities involving genetically modified organisms (GMOs) is presented in the law no. 11.105/2005, and in the normative Resolutions of the National Biosafety Technical Commission (CTNBio). CTNBio is responsible to advise the federal government on issues related to biosecurity of GMOs. One of the functions of CTNBio is evaluating whether there would be harm to non-target animals like bees before commercial release of GMOs.

Bees play an important role in pollination of wild and cultivated plants (Heard 1999; Kevan 1999; Klein et al. 2007; Winfree et al. 2007; Mitchell et al. 2009). Furthermore, bees also provide an important source of income and livelihood around the world (Kwapong et al. 2010; Formato and Smulders 2011; Devkota et al. 2016; dos Santos et al. 2021), especially for rural beekeepers. Since bees are dominant visitors to *Eucalyptus* flowers, where they obtain nectar and pollen in abundance (Heard 1999; Braga et al. 2012; Hilgert-Moreira et al. 2014; Gostinski et al. 2018), potential effects of novel substances inserted in these plants require risk assessments on bees. Such evaluation is critical, as bees appear to be sensitives to environmental contaminants. As result, they are good candidates for ecotoxicological analysis (Rose et al. 2007; Gill et al. 2012; Uhl et al. 2016; Cham et al. 2017; Krupke et al. 2017; Newhouse et al. 2021).

Among bees, the honey bee, *Apis mellifera* Linnaeus, 1758 stands out due to its wide geographic distribution and its ecological and economical value, being used as an experimental model in risk assessments of pesticides and GMOs (Malone and Pham-Delègue 2001; Desneux et al. 2007; Duan et al. 2008; Medrzycki et al. 2013; Sanchez-Bayo and Goka 2014; Ricroch et al. 2018). However, *A. mellifera* does not fully represent the diversity of bees in terms of susceptibility, which implies that native bees should be also explored in this context (Barbosa et al. 2015; Cham et al. 2017; Bernardes et al. 2018; Dorneles et al. 2021).

In the tropics, a wide diversity of social bees defined as stingless bees exist (Apidae: Meliponini) (Sakagami 1982). Recently, several species of stingless bees have been included in risk assessments of pesticides and other potential compounds like GMOs (Lima et al. 2013; Barbosa et al. 2015; dos Santos et al. 2016; Bernardes et al. 2018; Seide et al. 2018; Dorigo et al. 2019; Dorneles et al. 2021; Viana et al. 2022). The risk assessments with stingless bees is slightly distinctive from the one conducted with *A. mellifera*. Like honey bees, the stingless bees contribute to pollination in tropical agriculture (Heard 1999; Slaa et al. 2006) and for honey production, generating profitable income for smallholders (dos Santos et al. 2021). One prominent stingless bee species that has been selected as an indicator of the fauna of Brazilian native pollinators is *Scaptotrigona bipunctata* (Lepeletier, 1836). This species has populous colonies, with individuals adapted to experimental conditions (Dorneles et al. 2017, 2021; Diniz et al. 2021) and their natural geographic distribution has been registered in, at least, 13, out of 26 administrative Brazilian states (dos Santos et al. 2021).

To achieve a sustainable environment, risk assessments evaluate how the novel plants (genetically modified *Eucalyptus* trees) may affect non-target arthropods like bees. Thus, in order to contribute to the evaluation of a GMO event prior to its release for commercial use, the main goals of this study were to assess the survivorship (temporal attribute) and food consumption (behavioral attribute) of immatures and adults of two species of bees (*A. mellifera*, *S. bipunctata*) ingesting a diet containing pollen grains of genetically modified *Eucalyptus*.

## Materials and methods

### Bees

**Fig. 1:**
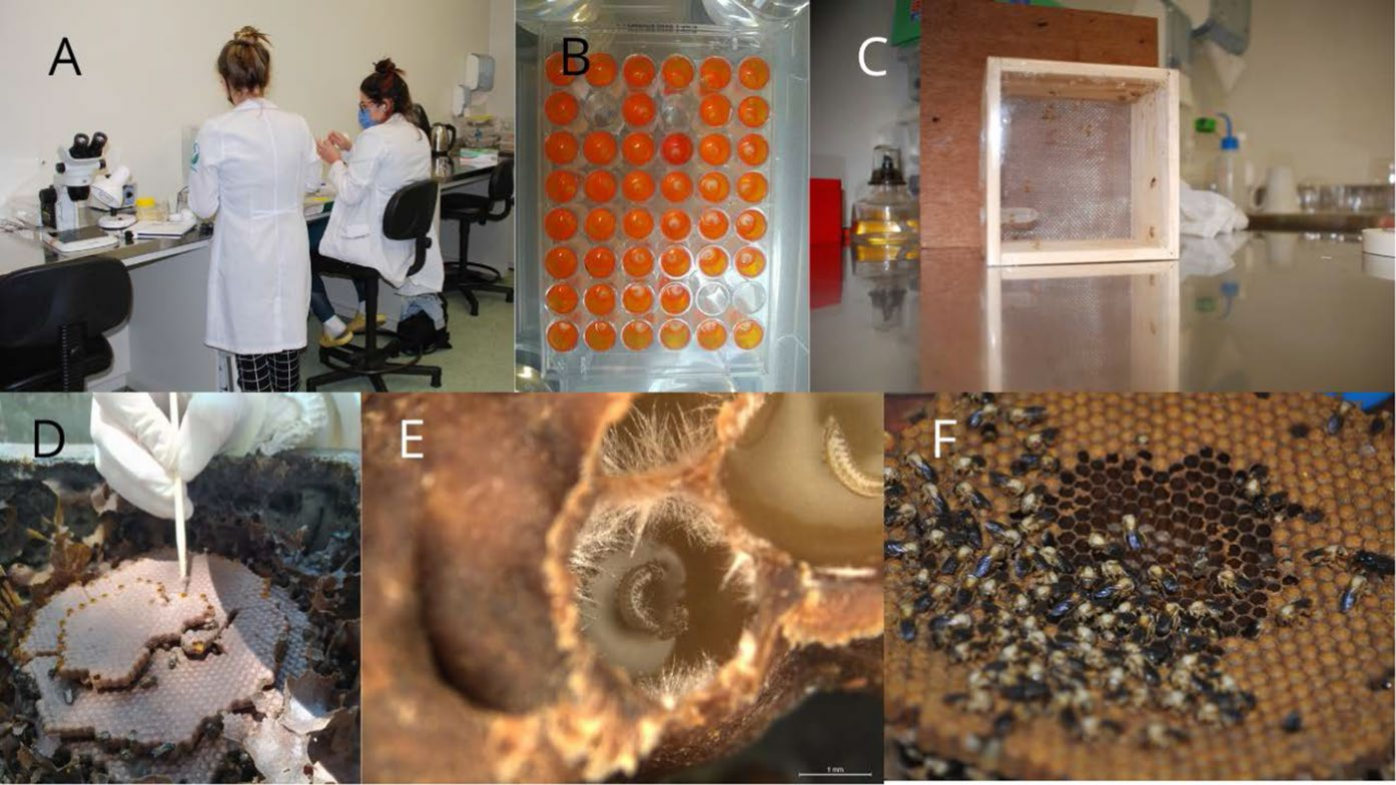
Pictures showing different moments of study. **A** Researchers (FGC, JDR) revising protocols prior conducting collects in beehives. **B** A rearing plate used to expose *Apis mellifera* (Apidae, Apini) larvae to different pollen diet. **C** A patterned wood box for exposing adults of *A. mellifera* to different pollen diets. **D** A comb of *Scaptotrigona bipunctata* (Apidae, Meliponini) used to provide bee larvae and larval food in the experiments. **E** A detail of two larvae of *S. bipunctata*. **F** Newly-emerged workers of *S. bipunctata* used for the experiments

Immature and adult bees came from standardized and healthy hives. Individuals of *A. mellifera* were obtained from three colonies kept in the apiary of the Federal University of Rio Grande do Sul (UFRGS). Specimens of the stingless bees *S. bipunctata* were obtained from three colonies maintained in the scientific stingless apiary (CTF475062/Cat20-45-MMA/IBAMA) of the Pontifical Catholic University of Rio Grande do Sul (PUCRS). Both locations, UFRGS and PUCRS, are located in the municipality of Porto Alegre, southern Brazil. All experiments were performed at Center for Experimental Biological Models (CeMBE, PUCRS) in an authorized biosafety structure (Biosafety Quality Certificate, CQB 390/15), Fig. 1A–E.

### Pollen

The pollen from *Eucalyptus* 751K032 (10 g), and conventional clone individuals FGN-K (10 g) were obtained from manual collections in the flowers within greenhouse cultivation of Suzano (FuturaGene Division), located in Itapetininga/ Brazil (CQB 325/11). Commercial, multifloral, dehydrated pollen grains (of honey bees) were included in treatments representing negative control. The choice of Eucalyptus 751K032 represents a landmark achievement in the world of agriculture and forestry, as it is the first genetically modified eucalyptus approved for commercial use that possesses tolerance to the herbicide glyphosate. This innovative technology offers a number of crucial benefits to the Brazilian forestry industry. It enables the implementation of effective and environmentally friendly weed control methods, eliminates the risk of damage to young plantlets from herbicide drift, leads to reduced operational costs and improved working conditions for forestry operators through the use of advanced machinery. In short, the adoption of this genetically modified eucalyptus event represents a major step forward in sustainable and efficient forestry management.

### Exposing immatures of *Apis mellifera* to different pollen diets

For exposures, (i) 10 mg of Eucalyptus pollen 751K032 was added in 30 µL of larval food (Steijven et al. 2016). Similarly, the control groups had the addition of, (ii) 10 mg of conventional *Eucalyptus* clone FGN-K (1st negative control) and (iii) 10 mg of multifloral pollen (2nd negative control) in 30 µL of larval food. The last treatment was (iv) diet without pollen addition (hereafter, WPA). The active ingredient dimethoate (99.3%, Ehrenstorfer GmbH) was used to evaluate the sensitivity of bees to toxic substances. It was added to the larval food, at the corresponding value of 8.8 µg a.i./larva for *A. mellifera* (OECD 2013).

*Apis mellifera* larvae were obtained from brood combs from three colonies, each one representing one replicate. Larvae aged 2 to 3 days were transferred to in vitro breeding plates and placed in breeding chambers with controlled temperature (32 ± 1 °C), relative humidity (95 ± 1%) and absence of light. The larvae were exposed to a single dose of the product on the 4th day of the test (OECD 2013). Each treatment (triplicate) was conducted with 12 bees. Therefore, there were 36 individuals per treatment, and a total of 144 honey bees were analyzed (triplicates × four treatments). After the onset of exposure, the survival rate was verified and recorded on 24 h, 48 h, and 72 h or, respectively, 5th, 6th and 7th days.

### Exposing immatures of *Scaptotrigona bipunctata* to different pollen diets

The amount of larval food offered to offspring of *S. bipunctata* within their natural colonies is 35 uL (Rosa et al. 2015) and the amount of pollen in such a volume of larval food is around 1.9 mg/ larva (Rosa et al. 2015). The treatments were composed by 35 µL aliquot of larval food with addition of (i) 1.9 mg of *Eucalyptus* pollen 751K032; (ii) 1.9 mg of conventional clone *Eucalyptus* FGN-K pollen (1st negative control); (iii) 1.9 mg of multifloral pollen (2nd negative control); and the fourth treatment was (iv) WPA. To evaluate the sensitivity of bees to toxic substances, 172 ng a.i./larva of the active ingredient dimethoate (99.3%, Ehrenstorfer GmbH) that is LD_50_ for *S. postica* (Dorneles et al. 2021) was added to the larval food.

*Scaptotrigona bipunctata* larvae were obtained from brood combs from three colonies, each one representing a replicate. Larvae aging 1 to 3 days were transferred to in vitro rearing plates. The feeding process for *S. bipunctata* larvae, like all stingless bees, is unique and distinct from that of *A. mellifera*. Unlike *A. mellifera*, where the larvae are fed progressively by nurse workers over several days, *S. bipunctata* larvae receive all the food they need in a single feeding. This method ensures that the larvae receive the proper nutrients in a timely manner to support their development (OECD 2013, 2017; Crailsheim et al. 2013), stingless bee species are fed massively, i.e., larvae receive the entire amount of food to develop to the adult stage (Sakagami 1982). Accordingly, *S. bipunctata* larvae were placed in breeding chambers with controlled temperature (28 ± 1 °C) and relative humidity (98 ± 1%) during the feeding period within following 1st to the 5th day. After that, saturated sodium chloride solution was added within in vitro rearing plates until the end of the experiment since it keeps relative humidity around 75% (Dorneles et al. 2021). Three replicates with 11 bees each were performed. Therefore, 33 individuals per treatment, totaling 132 stingless bees were analyzed (triplicates × four treatments). The survival of individuals was recorded daily until the 14th day, when they enter pre-pupa stage, with the larval development only.

### Exposing adults of *Apis mellifera* to different pollen diets

Mature brood combs of *A. mellifera* were collected from three colonies. The combs had no contact with each other. Each material was collected individually, the material was sterilized, and changed (when necessary) for each colony collection. Both the collection material and combs were individually placed in identified Petri dishes. They were kept in a brood chamber until adult emergence. The newly emerged workers, at a maximum of two days old, were transferred to wooden boxes (12 × 12 × 7 cm) for the tests. Prior to the beginning of the tests, the bees were adapted for 24 h, being fed with syrup only (1:1 sucrose solution). During the entire experiment, the bees were kept in rearing chambers with controlled temperature (32 ± 2 °C), relative humidity (70 ± 2%) and absence of light. Pollen was added to the syrup to facilitate consumption (Rose et al. 2007).

For exposing, the treatments were: (i) 13 mg of pollen of *Eucalyptus* 751K032 / mL of food (Yi et al. 2018); (ii) 13 mg of pollen from conventional clone *Eucalyptus* FGN-K (1st negative control); (iii) 13 mg of multifloral pollen (2nd negative control). Additionally, the active ingredient dimethoate (99.3%, Ehrenstorfer GmbH) CL_50_ for *A. mellifera*, which is 7.96 ng ai/µL of diet (Barbosa 2015) was evaluated to corroborate its toxicity to bees. Each experimental group was tested in triplicate, with 15 bees. Therefore, 45 individuals per treatment, totaling 135 honey bees were analyzed (triplicates × three treatments).

The bees were monitored daily in order to account for the mortality of individuals and the amount of diet consumed. The food was offered to the bees ad libitum during the exposure period of 10 days (chronic) and the food was changed daily. The parameters evaluated are in accordance with protocol 245—honey bee (*A. mellifera*), chronic oral toxicity test—10 day feeding (OECD 2017).

### Exposing adults of *Scaptotrigona bipunctata* to different pollen diets

Brood combs of *S. bipunctata* were collected from three colonies of the PUCRS stingless bee apiary. They were kept in a brood chamber until the emergence of adults. The newly emerged workers (max. 2 days) were collected and transferred to wooden boxes (12 × 12 × 7 cm) for testing. Prior to the beginning of the tests, the bees were adapted for 24 h, being fed with syrup only (1:1 sucrose solution). During the entire experiment, the bees were kept in rearing chambers with controlled temperature (28 ± 2 °C), relative humidity (70 ± 2%) and absence of light. Pollen was added to the syrup to facilitate consumption (Rose et al. 2007). The exposure treatments were identical for both honeybees and *S. postica*. Only that the sensitivity test using the CL_50_ of the active ingredient dimethoate, which was 3.24 ng ai/µL in the diet for *S. postica* (Tofili et al. 2018).

### Data analysis

All statistical analyzes were conducted in R programming language (Ihaka and Gentleman 1996; R Core Team 2021).

### *Eucalyptus *pollen 751K032 effect on immatures of *Apis mellifera*

The possible effects of *Eucalyptus* pollen 751K032on immatures of *A. mellifera* was analysed with a Chi-square test with multiple comparisons for each time point (24 h, 48 h, 72 h). For this, the ‘chisq.multpcomp’ function of the *RVAideMemoire* (Hervé 2020) was used. The *p*-values were adjusted with the Benjamini–Hochberg method.

### Effect of *Eucalyptus* pollen 751K032 on the survival of immatures and adults of honey bees and stingless bees

The evaluation of the possible effects of *Eucalyptus* pollen 751K032 on immatures of *S. bipunctata* and both adults of *A. mellifera* and *S. bipunctata* was performed by analyzing and comparing the survival curves for the larval development period of the immatures and for a pre-established period of consumption by the adults, respectively. In the first case, this period covered the first days of the larvae (1–3 days) until the phase in which they all became pupae, which is the most critical stage of bee development, where most mortality is commonly observed (Santos et al. 2015; Dorneles et al. 2021). In the second case, the exposure of adults occurred for a period of 10 consecutive days.

The Kaplan–Meier survival curves were generated using the ‘Surv’ function and the difference between them was tested using the ‘survdiff’ function both from the *survival* (Therneau 2020). Here, the development time (*right censored*) and the status, i.e., the event indicating 1 = death or 0 = survival of the organisms were evaluated by the treatments as predictor variable. This test was stratified by replicates to control for possible inconsistencies within each of them. Finally, the *log-rank* test was selected as a scalar parameter of the *G-rho family*, which prioritizes the differences at the end of two or more survival curves. After that, multiple comparisons were made between the survival curves to investigate which curves would be different according to the corresponding treatment. This procedure was done with *p*-values adjusted by the Benjamini–Hochberg method using the ‘pairwise_survdiff’ function of the *survminer* (Kassambara et al. 2020). The survival curve plots were constructed using the ‘survfit’ function from the *survival* (Therneau 2020), ‘ggsurvplot’ from the *survminer* (Kassambara et al. 2020) and *ggplot2* (Wickham 2016).

### *Eucalyptus* pollen effect 751K032 on food consumption by adults of honey bees and stingless bees

Finally, we performed repeated measures ANOVA to assess the daily food consumption by adults of *A. mellifera* and *S. bipunctata*. In this model, consumption was the response variable and treatments the predictor (fixed) variable, with the random variable assigned as the replicates per treatment. This analysis was done using the ‘aov’ function in R. Pairwise comparison was extracted with the ‘emmeans’ function of the *emmeans* (Lenth 2022) using Tukey method.

## Results

### Mortality of immatures of *Apis mellifera* after 24 h, 48 h and 72 h

**Fig. 2:**
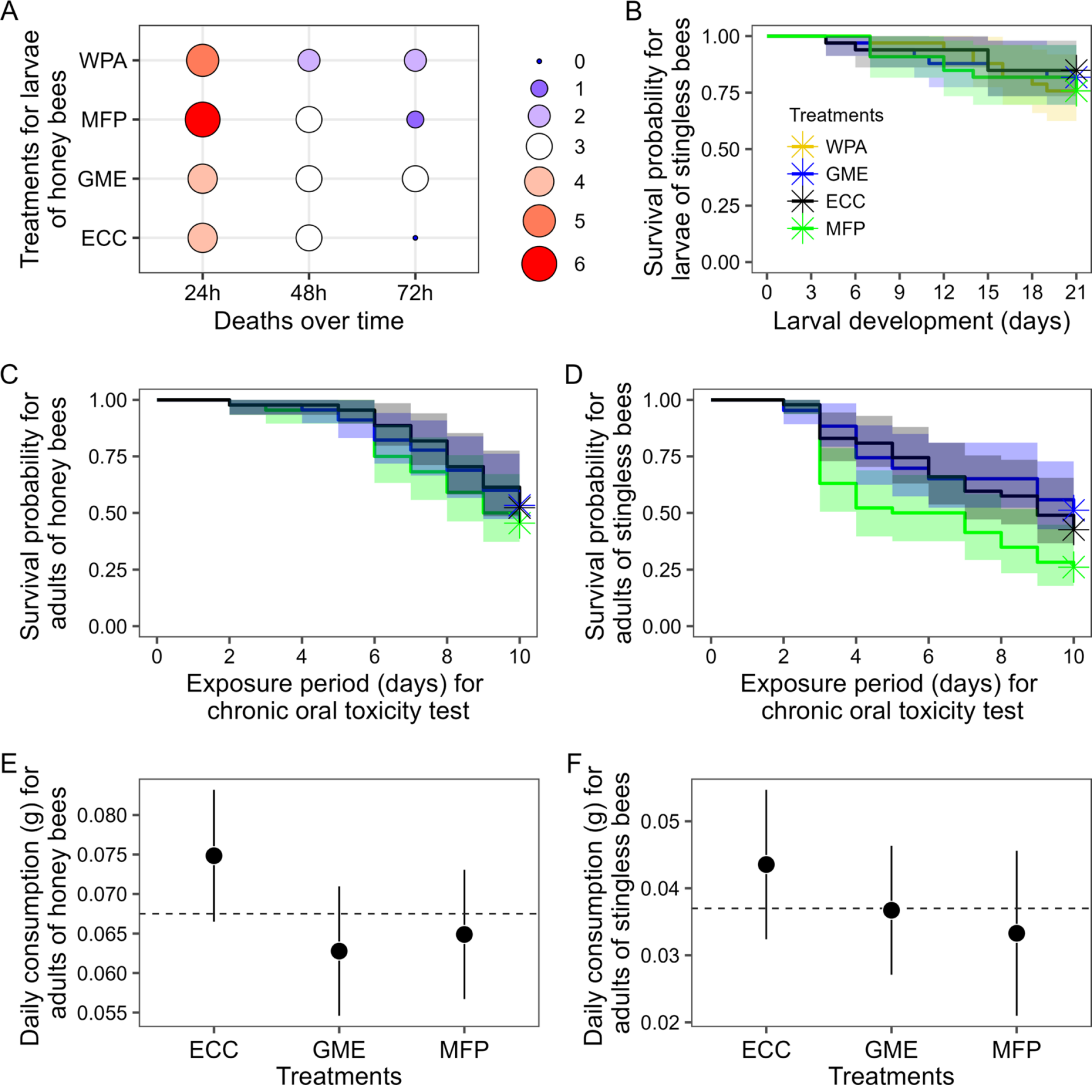
**A** Ballonplot showing results of Chi-square test. The size of points indicate the proportional mortality values of larvae of *Apis mellifera* (Apidae, Apini) exposed to different pollen diets. Note: The legend on the right shows the values in size scale and by color scale, the hottest being the biggest losses. The acronyms used in y-axis are applicable to panels **B** up to **F**, they are: WPA = diet without pollen addition; GME = *Eucalyptus* pollen 751K032; ECC = conventional clone eucalyptus pollen FGN-K [1st negative control]; MFP = Multifloral pollen [2nd negative control]. **B** Kaplan–Meier survival curves for larvae of *Scaptotrigona bipunctata* (Apidae, Meliponini). Notes: 95% confidence intervals are provided as colored shadows. Asterisks mean right censored (end of experiment). It is also applicable for panels **C** and **D**. **C** Kaplan–Meier survival curves for adults of *A. mellifera* exposed to different types of pollen diets. **D** Kaplan–Meier survival curves for adults of *S. bipunctata* exposed to different types of pollen diets. **E** and **F** Daily consumption of food offered to both adults of *A. mellifera* and *S. bipunctata*, respectively, containing different pollen diets. Notes: Points mean average values, while vertical lines indicate 95% confidence intervals; Horizontal dashed lines show the overall average of consumption. It was inserted to facilitate interpretation. (Color figure online)

Practically all *A. mellifera* immatures exposed to dimethoate died in the first hours of observation. Yet, the deaths under statistical analysis of immatures of *A. mellifera* during the experiments of exposure to different treatments through the diet showed no significant difference (Table 1, Fig. 2A). Therefore, the groups treated with *Eucalyptus* pollen 751K032 and with the other diets did not differ significantly.

**Table 1:** Results of the Chi-square test with paired comparisons on the mortality of immatures of *Apis mellifera* (Hymenoptera: Apidae, Apini) treated with different diets, including eucalyptus pollen 751K032. The acronyms given in the treatments column are the same as those used in paired comparisons

Periods	χ^2^	Degrees of freedom	*p*-values
24 h	0.57	3	0.90
48 h	0.27	3	0.96
72 h	3.33	3	0.34

	WPA	GME	ECC
*Pairwise comparisons *(*p-values*)			
**24 h**			
GME	1.00	–	–
ECC	0.92	0.92	–
MFP	0.92	0.92	0.92
**48 h**			
GME	1.00	–	–
ECC	1.00	1.00	–
MFP	1.00	1.00	1.00
**72 h**			
GME	0.48	–	–
ECC	0.47	0.65	–
MFP	0.47	0.48	0.65

WPA = diet without pollen addition; GME = eucalyptus pollen 751K032; ECC = conventional clone eucalyptus pollen FGN-K; MFP = multifloral pollen

### Survival curves of immatures of *Scaptotrigona bipunctata*

The immatures of *S. bipunctata* did not survive for more than five days after exposure to dimethoate. On the other hand, data indicate that there was no difference between treatments using any types of pollen and the diet without pollen addition; however (χ^2^ = 1.00, *p*-value = 0.80, Table 2, Fig. 2). Treatments using pollen from *Eucalyptus* 751K032 and conventional clone *Eucalyptus* FGN-K tended to have a slightly higher survival (>=81%) than the control without pollen addition (75%), although this difference is not significant (Table 2; Fig. 2B).

**Table 2:** Results of survival curve analysis by log-rank test (upper) and test of pairwise comparisons between each treatment of the experiments of possible effects of eucalyptus pollen 751K032 on immature *Scaptotrigona bipunctata* (Apidae, Meliponini). The acronyms given in the treatments column are the same as those used in pairwise comparisons

Treatments	The amount	Observed	Expected	(OE)^2/E	(OE)^2/V
WPA	33	8	6.94	0.16	0.22
GME	33	5	6.70	0.43	0.58
ECC	33	6	6.74	0.08	0.11
MFP	33	8	6.62	0.28	0.38
	χ^2^ = 1.00, degrees of freedom = 3, *p*-value 0.80				

	WPA	GME	ECC
*Pairwise comparisons (p-values)*			
GME	0.88	–	–
ECC	0.88	0.88	–
MFP	0.95	0.88	0.88

WPA = diet without pollen addition; GME = eucalyptus pollen 751K032; ECC = conventional clone eucalyptus pollen FGN-K; MFP = multifloral pollen

### Survival curves of adults of both *Apis mellifera* and* Scaptotrigona bipunctata*

No adult *A. mellifera* exposed to dimethoate survived for more than two days. The survival curves of *A. mellifera* adults showed no difference between treatments with *Eucalyptus* pollen 751K032, conventional clone *Eucalyptus* pollen FGN-K and multifloral pollen (χ^2^ = 1.2, *p*-value = 0.50, Table 3, Fig. 2C). In descending order, the survival of *A. mellifera* adults was: *Eucalyptus* pollen 751K032 (53%), multifloral pollen (52%), conventional clone *Eucalyptus* pollen FGN-K (45%), Fig. 2C.

**Table 3:** Results of survival curve analysis by log-rank test (upper) and test of pairwise comparisons between each treatment of the experiments of possible effects of eucalyptus pollen 751K032 on adult worker bees of *Apis mellifera* (Hymenoptera: Apidae, Apini). The acronyms given in the treatments column are the same as those used in pairwise comparisons

Treatments	The amount	Observed	Expected	(OE)^2/E	(OE)^2/V
GME	45	21	23.0	0.17	0.29
ECC	44	24	20.1	0.74	1.20
MFP	44	21	22.9	0.15	0.26
	χ^2^ = 1.2, degrees of freedom = 2, *p*-value = 0.50				

	GME	ECC
*Pairwise comparisons* (*p-values*)		
GME	0.64	–
MFP	0.64	0.96

GME = eucalyptus pollen 751K032; ECC = conventional clone eucalyptus pollen FGN-K; MFP = multifloral pollen

Similarly, no adult *S. bipuctata* exposed to dimethoate survived for more than four days. The survival curves of *S. bipunctata* adults indicate differences between treatments (χ^2^ = 7.3, *p*-value < 0.03, Table 4, Fig. 2D). In the pairwise analysis, conventional clone *Eucalyptus* FGN-K pollen differed of *Eucalyptus* pollen 751K032, Table 4. In this case, the survival rate of adults fed with conventional pollen was practically half of that observed for bees treated with *Eucalyptus* pollen 751K032. However, there was no difference between *Eucalyptus* pollen 751K032 and multifloral pollen, Table 4. In descending order, the survival of *S. bipunctata* adults was: *Eucalyptus* pollen 751K032 (51%), multifloral pollen (42%), and conventional clone *Eucalyptus* FGN-K pollen (26%), Fig. 2D.

**Table 4:** Results of the survival curve analysis by the log-rank test (upper) and the test of pairwise comparisons between each treatment of the experiments of the possible effects of 751K032 eucalyptus pollen on adult worker bees of *Scaptotrigona bipunctata* (Hymenoptera: Apidae, Meliponini). The acronyms given in the treatments column are the same as those used in pairwise comparisons

Treatments	The amount	Observed	Expected	(OE)^2/E	(OE)^2/V
GME	43	21	28.6	2.00	3.53
ECC	46	34	24.1	4.11	6.80
MFP	47	27	29.4	0.19	0.35
	χ^2^ = 7.30, degrees of freedom = 2, *p*-value < 0.03				

	ECC	GME
*Pairwise comparisons* (*p-values*)		
GME	0.02	–
MFP	0.06	0.50

GME = eucalyptus pollen 751K032; ECC = conventional clone eucalyptus pollen FGN-K; MFP = multifloral pollen

### Food consumption by adults of both *Apis mellifera* and *Scaptotrigona bipunctata*

There was no difference in food consumption by *A. mellifera* adults (*repeated measures* ANOVA, F_(2, 73)_ = 2.47, *p*-value = 0.09), Table 5, Fig. 2E). The same was observed, that is, no difference in food consumption by adults of *S. bipunctata* (*repeated measures* ANOVA, F_(2, 67)_ = 0.83, *p*-value = 0.43). Evidently, therefore, in the pairwise comparisons there are no differences between the pairs of treatments (Table 6, Fig. 2F).

**Table 5:** Results of the repeated measures ANOVA showing the paired comparisons on the daily food consumption by adult bees of *Apis mellifera* (Hymenoptera: Apidae, Apini) related to the experiments of possible effects of eucalyptus pollen 751K032 on these bees. The acronyms given in the treatments column are the same as those used in pairwise comparisons with Tukey method

	Degrees of freedom	Sum of squares	F-value	*p*-value
Treatments	2	0.002	2.47	0.09
Residuals	73	0.029		

	Estimate	SE	*p*-values
*Pairwise comparisons*			
GME × ECC	0.012	0.005	0.09
ECC × MFP	0.009	0.005	0.19
GME × MPF	−0.002	0.005	0.92

GME = eucalyptus pollen 751K032; ECC = conventional clone eucalyptus pollen FGN-K; MFP = multifloral pollen; SE = Standard Error

**Table 6:** Results of the repeated measures ANOVA showing the paired comparisons on the daily food consumption by adult bees of *Scaptotrigona bipunctata* (Hymenoptera: Apidae, Meliponini) related to the experiments of the possible effects of eucalyptus pollen 751K032 on these bees. The acronyms given in the treatments column are the same as those used in pairwise comparisons with Tukey method

	Degrees of freedom	Sum of squares	F-value	*p*-value
Treatments	2	0.001	0.83	0.43
Residuals	67	0.045		

	Estimate	SE	*p*-value
*Pairwise comparisons*			
GME × ECC	0.006	0.007	0.66
ECC × MFP	0.009	0.007	0.41
GME × MPF	0.003	0.007	0.90

GME = eucalyptus pollen 751K032; ECC = conventional clone eucalyptus pollen FGN-K; MFP = multifloral polle; SE = Standard Error

## Discussion

The findings suggest that the survivorship and the food consumption of immatures and adults of *A. mellifera* and *S. bipunctata* were not affected by genetically modified eucalyptus pollen (*Eucalyptus* pollen 751K032). Overall, the treatments with *Eucalyptus* pollen 751K032 and conventional clone *Eucalyptus* and multifloral pollen (negative controls) demonstrated very similar survival curves and food consumption, according to the bee species being tested. Subsequently, as food offered to larvae included a diet without pollen addition, results were concordant with other treatments (novel event and negative controls). Parallel, it was observed that most immatures and adults of both bee species exposed to dimethoate, a well-known toxic insecticide for bees (Crailsheim et al. 2013; Pirk et al. 2013), died faster than in treatments under statistical analysis or they tended to consume an extremely low amount of food, if any.

We highlight that despite the fact that these newly expressed proteins are known to be non-toxic to many insect species, including bees, it was necessary to perform bioassays to exclude possible unintended effects. This approach is consistent with regulatory requirements in Brazil and EU legislation, as well as scientific best practices since it provides important information for stakeholders about safety and potential benefits for pollinators.

Commonly, risk assessment studies structure their experimental designs with one positive control and a single negative control (Malone and Pham-Delègue 2001; Desneux et al. 2007; Duan et al. 2008; Crailsheim et al. 2013; Medrzycki et al. 2013; Pirk et al. 2013; Sanchez-Bayo and Goka 2014). Here, in order to augment our decision power in inferring whether the novel substance under evaluation (*Eucalyptus* pollen 751K032) might or might not cause adverse effects on the bees being evaluated, the experiments were carried out with two negative controls, namely the conventional clone *Eucalyptus* FGN-K (1st negative control) and the multifloral pollen (2nd negative control). Consequently, suitable and more consistent evidence demonstrated that the target GMO had no effect during the development larval of *A. mellifera* and *S. bipunctata*. Similarly, adults of both bee species showed equivalent rates of food intake.

The major goal of these experiments was to compare the protein diet containing *Eucalyptus* pollen 751K032 against mainly conventional clone *Eucalyptus* FGN-K and multifloral pollen. As they were more similar to each other exhibiting average values and confidence intervals close to the overall mean, all treatments with any type of pollen were congruent per se.

To add more reliability in the risk assessment, this study was carried out with two bee species. Therefore, not only a traditional bee specie was tested, i.e., *A. mellifera* (Malone and Pham-Delègue 2001; Duan et al. 2008; Romeis et al. 2008, 2011; Hilbeck et al. 2011; Arpaia et al. 2021), but also a native and surrogate stingless bee species (*S. bipunctata*), as recommended by Brazilian norms (Cham et al. 2017). Also, different pollen diets were offered and they were evaluated in two stages of the life cycle of both bee species (immatures and adult workers). Finally, survivorship was investigated for individuals over time during their larval development as well as survival of adults consuming different pollen diets. Aggregating the results, it may be considered that the genetically modified eucalyptus pollen (*Eucalyptus* pollen 751K032) is harmless to the organisms assessed.

The measurement of food consumption is only one aspect of the numerous sublethal effects that are relevant to pollinators (Arpaia et al. 2011). This is why a more comprehensive approach is needed to understand the potential impact of genetically modified agro-ecosystems on bee populations. A multi-faceted approach, including examination of reproduction, behavior, and overall health, provides a more comprehensive understanding of the potential impact on pollinators. For a sustainable environment, it is important to constantly review the exposure of bees to genetically engineered crops (Seide et al. 2018). However, recent literature reviews have indicated that most of GM crops are harmless to bees under laboratory conditions or in field/semi-field trials (Ricroch et al. 2018; Arpaia et al. 2021). Accordingly, this work which demonstrated a lack of adverse effects on two bee species during the ingestion by immatures and adults of the *Eucalyptus* pollen 751K032, adds to this growing body of safety data.

Based on a comprehensive analysis of previous research on the impact of GM crops on pollinators, studies have shown that the impact on bees and other pollinators has been either neutral or minimal. Thus, although some studies have found limited evidence of a significant impact on bee health, the negligible effects find of GM crops and outcomes from lab, semi-field, and field on bees cannot be overemphasized. It is imperative to continue the discussion and further investigate the effects of GM crops on pollinators, which is crucial in making well-informed decisions about their use in agriculture and ensuring the sustainable health of pollinator populations.

## Data Availability

The datasets generated and analyzed during the current study are deposited in GitHub repository in Zenodo.
